# Supplementary material for: Economic violence against women: A case in Turkey
Source: PLoS One. 2021 Mar 15;16(3):e0248630. doi: 10.1371/journal.pone.0248630 (PMC7959383; doi:10.1371/journal.pone.0248630)
Supplement: Attachment — Submitted filename: Review_Economic Violence against Women A case in Turkey.docx [file pone.0248630.s001.docx]

Comments on the Manuscript: **Economic Violence against Women: A case in Turkey**

The subject of manuscripts appears interesting, but some important revisions for clarity are needed before publication decision.

The hypothesis of the research are not clear. The author(s) needs to explain what are the main research questions of this paper. This paper needs a comprehensive literature review on the issue of conceptualization of economic violance. The hypothesis address this issue and the main contribution to literature should be explained.

It is sufficient to present ratios for descriptive statistics. It is not clear why the author(s) apply Chi-square tests such as the Chi-square test between Survey year and “Exposure to Economic Violence”.

The paper use “The binary logistic regression” methodology. It present “odd ratios”. But, it could be better if the author(s) calculates “marginal effects” and add them to the manuscript.

The manuscript includes unnecessary explanations about Tables. For example Table 3 includes odds ratios and confidence intervals. However, the following paragraphs repeat all these statistics. It will be better to explain the summary of the results and their connection with the hypothesis.

The dependent variable is created with respect to answers of the women about their husband/partner’s behaviour. So, it seems that the research includes women who live with their husbands/partners. Some of the explanations lose its meaning such as “The women who had a relationship and were married had a higher odds ratio of exposure to economic violence compared to women who had no existing relationship.” On the other hand, the data source of the research is limited. The subject in this research needs to collect data using interviews with women. For example Sen, S. and Bolsoy, N (2017) collect data using questionnaires and “Scale of Domestic Violence against Women”.

There are some contradictions in the manuscript. For example, in the abstract, the explanation about education level as follow: “Women who graduated from elementary school, secondary school, and high school had a higher odds ratio of exposure to economic violence compared to those who have never gone to school.” P.19 “Women who graduated from elementary school, secondary and high school had higher possibility of exposure to economic violence by 18.34%, 25.14% and 19.4%, respectively, compared to women who have never finished school.”

However P.21 there is the following explanation: “In the study, it was determined that the educational status of women had an effect on exposure to economic violence. In the study, it was concluded that the possibility of exposure to economic violence decreased as the educational level increased.”

The author(s) claims that the paper can be a significant guide in determining the priority areas for the solution of economic violence. However, the connections between the results of research and priority area explanations in the conclusion are weak. It needs to explain relations between the results and priority area explanations.

The authors also needs to read the text carefully and should correct grammatical mistakes. It needs to proof reading before to submitting.
