# Supplementary material for: Economic violence against women: A case in Turkey
Source: PLoS One. 2021 Mar 15;16(3):e0248630. doi: 10.1371/journal.pone.0248630 (PMC7959383; doi:10.1371/journal.pone.0248630)
Supplement: Attachment — Submitted filename: Response to reviewers.docx [file pone.0248630.s002.docx]

**Additional Editor Comments (if provided):**

Both referees read the paper with interest but share the view that substantive changes are necessary before the paper can be considered for publication. I have also gone through the paper and share the referee recommendations. Referee 2 has emphasized on the need to engage better with the literature and be more critical in discussing the key concepts & measures (e.g. women’s exposure to economic IPV) and provided a detailed revision guideline. Other recommendations include a thorough language check to correct grammatical mistakes.

I hope all of these addressed during the revision stage. I also have a few other points for the authors to consider.

**1. Comment:** In the discussion section (2nd last para), the authors say: "Women's empowerment, and to consider them as individuals existing in society are important steps to prevent violence. Unless a society that understands that women can have economic potential and take part in successful jobs is formed, violence against women should not be expected to end completely." Pls avoid such unsubstantiated claims and viewpoints. There's already a literature on the link between empowerment and violence. Kindly cite relevant studies in support of your claim.

**1. Response:** In line with the editor's criticism, the relevant paragraph has been removed from the paper.

**2.** **Comment:** tables 3-4: pls report sample size. also no need to report confidence intervals

**2. Response:** Table 3-4 has been reorganized and confidence intervals have been removed. The sample volume is also indicated in the table.

**3.** **Comment:** the paper would also benefit from a section on study background and context so that readers unfamiliar with Turkey has an introduction to norms of violence, gender roles and power balance within marriage in Turkey.

**3. Response:** The necessary correction has been made in accordance with the editor's recommendation. For this purpose, the title *“Economic violence/abuse and gender roles in Turkey”* was added into the introduction section.

**Reviewers' comments:**

**Reviewer #1:**

The subject of manuscripts appears interesting, but some important revisions for clarity are needed before publication decision.

**1. Comment:** The hypothesis of the research are not clear. The author(s) needs to explain what are the main research questions of this paper. This paper needs a comprehensive literature review on the issue of conceptualization of economic violence. The hypothesis address this issue and the main contribution to literature should be explained.

**1. Response:** In accordance with the criticism of the Reviewer, the main research questions of the paper were added to the introduction section. A comprehensive literature research was carried out and the title *“Literature review”* was added into the paper.

**2. Comment:** It is sufficient to present ratios for descriptive statistics. It is not clear why the author(s) apply Chi-square tests such as the Chi-square test between Survey year and “Exposure to Economic Violence”.

**2. Response:** The necessary corrections were made in accordance with the criticism of the Reviewer. The survey year variable is removed from the chi-square analysis Table. Necessary explanations have been made about the chi-square test. In accordance with the criticism, the description *“Bivariate analyses were also performed to identify relationships between the outcome variable (exposure to economic violence) and various factors. We estimated bivariate relationships by evaluating significant differences using Pearson chi-square tests for categorical variables. Pearson chi-square (χ2) not only provides information about the importance of observed differences, but also provides detailed information about which categories any differences found arise from”* was added into the paper.

**3. Comment:** The paper use “The binary logistic regression” methodology. It present “odd ratios”. But, it could be better if the author(s) calculates “marginal effects” and add them to the manuscript.

**3. Response:** In accordance with the Reviewer's criticism, “odd ratios” comments were removed and marginal effects were interpreted in the paper.

**4. Comment:** The manuscript includes unnecessary explanations about Tables. For example Table 3 includes odds ratios and confidence intervals. However, the following paragraphs repeat all these statistics. It will be better to explain the summary of the results and their connection with the hypothesis. The dependent variable is created with respect to answers of the women about their husband/partner’s behavior. So, it seems that the research includes women who live with their husbands/partners. Some of the explanations lose its meaning such as “The women who had a relationship and were married had a higher odds ratio of exposure to economic violence compared to women who had no existing relationship.” On the other hand, the data source of the research is limited. The subject in this research needs to collect data using interviews with women. For example Sen, S. and Bolsoy, N (2017) collect data using questionnaires and “Scale of Domestic Violence against Women”.

**4. Response:** Table 3 comments were revised in accordance with the Reviewer's criticism. In accordance with the criticism, the results have been reinterpreted. In the paper, our explanation on the dependent variable was insufficient. *“Has your spouse or any of the people you have been with ...”* has been added into the paper regarding the dependent variable. Additional explanations were made about the "relationship status" variable in the paper. The phrase *“In this study, women who were married, had a relationship or had any previous relationship were included in the analysis. Single women who had never been in a relationship before were not included in the study.”* was added to the paper. Secondary data was used in the paper. *“The National Research on Domestic Violence against Women in Turkey”* data was used with official permission from the Turkish Statistical Institute. *“In order to understand and determine the causes the different dimensions of violence against women, and also to fulfill the need to collect data on this subject, for the first time in 2008, a comprehensive "The National Research on Domestic Violence against Women in Turkey " was held. " The National Research on Domestic Violence against Women in Turkey" which was carried out in 2014 is important in terms of reflecting the violence against women during the time change. “The National Research on Domestic Violence against Women in Turkey” is one of the most comprehensive studies conducted nationwide in order to understand the extent, content, causes and consequences of domestic violence experienced by women, as well as the risk factors”* explanation has been added into the paper about the reason why this dataset is used.

**5. Comment:** There are some contradictions in the manuscript. For example, in the abstract, the explanation about education level as follow: “Women who graduated from elementary school, secondary school, and high school had a higher odds ratio of exposure to economic violence compared to those who have never gone to school.” P.19 “Women who graduated from elementary school, secondary and high school had higher possibility of exposure to economic violence by 18.34%, 25.14% and 19.4%, respectively, compared to women who have never finished school.” However P.21 there is the following explanation: “In the study, it was determined that the educational status of women had an effect on exposure to economic violence. In the study, it was concluded that the possibility of exposure to economic violence decreased as the educational level increased.”

**5. Response:** In accordance with the criticism of the Reviewer, the contradiction in Page 19 and Page 21 in the paper was corrected and written accordingly.

**6. Comment:** There The author(s) claims that the paper can be a significant guide in determining the priority areas for the solution of economic violence. However, the connections between the results of research and priority area explanations in the conclusion are weak. It needs to explain relations between the results and priority area explanations.

**6. Response:** The discussion section was reorganized in accordance with the criticism of the Reviewer. The results found are re-discussed in detail.

**7. Comment:** The authors also needs to read the text carefully and should correct grammatical mistakes. It needs to proof reading before to submitting.

**7. Response:** Language and grammatical errors were corrected during the “proofreading” made by the editor, whose mother tongue is English, in accordance with the criticism of the Reviewer.

**Reviewer #2:**

This study examined the correlates of women’s exposure to economic coercion in Turkey using the micro data set of the National Research on Domestic Violence against Women in Turkey. The topic of women’s exposure to economic coercion is salient and understudied, so this manuscript makes an important empirical contribution that is worthy of publication and that will be of interest to researchers of violence against women and women’s health. Below are suggestions that the authors might consider to improve the contributions that this manuscript makes.

Introduction/Background

**1. Comment:** The literature review should be strengthened considerably by including some of the seminal conceptual and empirical contributions to the field. For example, the authors may consider citing the following studies (there are many others, so these are illustrative):

**1. Response:** In accordance with the criticism of the Reviewer, a comprehensive literature research was carried out and the title *“Literature review”* was added into the paper. The studies recommended by the Reviewer were reviewed in detail and the introduction section was rewritten to be used in the paper.

**2. Comment:** Conceptualization and reviews of economic coercion/IPV. Here, the authors should offer a clear definition of economic IPV/coercion. What are the dimensions of economic IPV, as discussed in the literature? Providing a clear definition will allow the authors to critique how well economic IPV is measured in their data source (see comment about the discussion).

**2. Response:** In accordance with the criticism of the Reviewer, the concept of economic violence is explained in detail in the introduction section. The definition of economic violence, its dimensions and how economic violence is measured in the data source are explained in detail.

**3. Comment:** The authors should provide more detail on the data source. What were the inclusion criteria? Was this a cross-sectional study? What was the sampling strategy? What was the response rate?

**3. Response:** Detailed explanations on the data source were written in accordance with the criticism of the Reviewer. Accordingly, phrases *“As part of the Violence Survey, Turkey is divided into 30 layers to provide estimations at the level of Country, urban/rural, 12 regions and 5 regions. Except for the Istanbul region, which is one of the 12 regions, approximately 75 percent and 25 percent distribution was made to urban and rural layers. In Istanbul, about 5 percent of households were selected from rural. In the study, settlements with a population of 10,000 and above constitute urban areas, and settlements with a population of less than 10,000 constitute rural areas. The sample of the research is cluster sampling”*, *“In a 2008 study, 12,795 women were interviewed face-to-face and the female question paper was completed with a rejection rate of 2.1%. The answer rate in female interviews is 86.1%. In the 2014 survey, 7,462 women were interviewed face-to-face and the female question paper was completed, with a rejection rate of 4.4%. The answer rate in female interviews is 83.3%. These data sets included female weights calculated to match the sample design of the study”*, and *“In this study, women who were married, had a relationship or had any previous relationship were included in the analysis. Single women who had never been in a relationship before were not included in the study. In conclusion, the data of a total of 18,225 women aged 15 years and over who participated in the National Research on Domestic Violence against Women in Turkey, including 11,514 women in 2008 and 6,711 women in 2014, were employed”* were added into the paper.

**4. Comment:** The authors also should provide a critical discussion of the three questions on economic IPV? How well do they align with definitions of economic coercion/IPV? What aspects of economic coercion/IPV are captured? What are missed? What was the time frame for which participants were asked to report on experiences of economic IPV—lifetime? Prior year? Why did the authors select a binary outcome instead of a count outcome (number of types of economic coercion experienced in the given time frame?)

**4. Response:** The aspects of economic violence have been rewritten in accordance with the criticism of the Reviewer. New information on questions related to economic violence in data source is provided. The time frame for economic violence is written in more detail. Extra explanations are provided related to why the dependent variable is qualitative.

**5. Comment:** The authors say that they relied on the literature for decisions about selections for the correlates of IPV, but do not cite any literature in the discussion of correlates. I refer the authors here to the article by Yount et al. in Vietnam as a relevant reference in this section.

**5. Response:** A comprehensive literature research was conducted in accordance with the criticism of the Reviewer. The study recommended by the Reviewer was examined in detail and used in the paper.

**6. Comment:** Analytically, I wonder about the extent to which other forms of violence “predict” economic coercion, or whether these are co-occurring and reciprocally influential behaviors in an abusive relationship. The temporal ordering of the variables is critical, but is not well described. I encourage the authors to be more transparent about the timeframe for which different forms of violence are measured, and the temporal ordering of economic coercion with other forms of IPV used as predictors.

**6. Response:** Detailed explanations on other types of violence have been added in accordance with the criticism of the Reviewer. As a result of literature research, the relationship between economic violence and other types of violence has been extensively discussed.

**7. Comment:** The authors should do a better job of critiquing the measure of women’s exposure to economic IPV. What are the implications of poor measurement for inferences from the analysis? What recommendations come out of the limitations of the measure of economic IPV used in the study?

**7. Response:** In accordance with the criticism of the Reviewer, the discussion section was reorganized and the necessary explanations were added.
