# Supplementary material for: Economic violence against women: A case in Turkey
Source: PLoS One. 2021 Mar 15;16(3):e0248630. doi: 10.1371/journal.pone.0248630 (PMC7959383; doi:10.1371/journal.pone.0248630)
Supplement: Attachment — Submitted filename: Referee report.docx [file pone.0248630.s003.docx]

**Referee Report for Manuscript No. PONE-D-20-15042_R1 “Economic Violence against Women: A case in Turkey”**

I am reviewing authors’ edits to the manuscript and their responses to the previous reviewer #1’s comments. I am happy to see that the authors have addressed all the comments that reviewer #1 has raised and the manuscript is significantly improved. Specifically, the manuscript adds a comprehensive literature review, modifies discussions on “marginal effects” and Pearson chi-square tests, and explains tables better. The contradictory results are corrected, and the discussion section was reorganized.
